# A protocol to isolate, identify, and verify glucose- or carbohydrate-binding receptors

**DOI:** 10.1093/biomethods/bpae045

**Published:** 2024-06-19

**Authors:** Nadia Rashid, Kavaljit H Chhabra

**Affiliations:** Department of Pharmacology and Nutritional Sciences, University of Kentucky, Lexington, KY 40506, United States; Barnstable Brown Diabetes and Obesity Research Center, University of Kentucky, Lexington, KY 40506, United States; Department of Pharmacology and Nutritional Sciences, University of Kentucky, Lexington, KY 40506, United States; Barnstable Brown Diabetes and Obesity Research Center, University of Kentucky, Lexington, KY 40506, United States

**Keywords:** glucose/carbohydrate receptors, affinity chromatography, In-cell ELISA, surface plasmon resonance (SPR), diabetes, obesity, metabolism

## Abstract

Sensing, transport, and utilization of glucose is pivotal to the maintenance of energy homeostasis in animals. Although transporters involved in mobilizing glucose across different cellular compartments are fairly well known, the receptors that bind glucose to mediate its effects independently of glucose metabolism remain largely unrecognized. Establishing precise and reproducible methods to identify glucose receptors in the brain or other peripheral organs will pave the way for comprehending the role of glucose signaling pathways in maintaining, regulating, and reprogramming cellular metabolic needs. Identification of such potential glucose receptors will also likely lead to development of effective therapeutics for treatment of diabetes and related metabolic disorders. Commercially available biotin or radiolabeled glucose conjugates have low molecular weight; therefore, they do not provide enough sensitivity and density to isolate glucose receptors. Here, we describe a protocol to isolate, identify, and verify glucose-binding receptor/s using high molecular weight glucose (or other carbohydrate) conjugates. We have produced 30 kDa glucose– (or other carbohydrate–) biotin–polyacrylamide (PAA) conjugates with mole fractions of 80:5:15% respectively. These conjugates are used with biotin-streptavidin biochemistry, In-cell ELISA, and surface plasmon resonance (SPR) methods to isolate, identify, and verify glucose- or carbohydrate-binding receptors. We first demonstrate how streptavidin-coated magnetic beads are employed to immobilize glucose–biotin–PAA conjugates. Then, these beads are used to enrich and isolate glucose-binding proteins from tissue homogenates or from single-cell suspensions. The enriched or isolated proteins are subjected to mass spectrometry/proteomics to reveal the identity of top candidate proteins as potential glucose receptors. We then describe how the In-cell ELISA method is used to verify the interaction of glucose with its potential receptor through stable expression of the receptor *in-vitro*. We further demonstrate how a highly sensitive SPR method can be used to measure the binding kinetics of glucose with its receptor. In summary, we describe a protocol to isolate, identify, and verify glucose- or carbohydrate-binding receptors using magnetic beads, In-cell ELISA, and SPR. This protocol will form the future basis of studying glucose or carbohydrate receptor signaling pathways in health and in disease.

## Introduction

Binding of a ligand molecule to its receptor is specific in nature [1]. Carbohydrates are a diverse group of molecules known to modulate specific drug targets such as G protein-coupled receptors (GPCRs) [2]. Glucose-binding proteins and their potential signaling pathways have been described in plants, bacteria, and fungi [3–5]. However, isolation, identification, and characterization of such glucose- or carbohydrate-binding receptors have been elusive in animals because of the lack of suitable reagents and tools. Identifying glucose receptors has the potential to unravel glucose signaling and sensing pathways in addition to developing drug targets for treating metabolic diseases such as diabetes mellitus.

The isolation of glucose receptors has been challenging because currently available glucose conjugates such as biotin- or radio-labeled glucose reagents do not provide sufficient sensitivity and density for an affinity chromatography assay owing to their low molecular weight as described in our previous publication [6]. To overcome this limitation, we developed a protocol to isolate glucose- or carbohydrate-binding receptors using a high molecular weight 30 kDa glucose– (or other carbohydrate–) biotin–polyacrylamide (PAA) conjugate with mole fractions of 80:5:15% respectively. We first show how the 30 kDa glucose– or galactose–biotin–PAA conjugates can be captured using streptavidin-coated magnetic beads. We then use concanavalin A or jacalin, which are known carbohydrate-binding proteins, to exemplify how glucose- or galactose-binding receptor/s can be retrieved and enriched from tissue homogenates or from single-cell suspensions. Carbohydrate-binding proteins are called lectins which exhibit diverse biological functions based on their different sugar-binding affinities [7]. Concanavalin A is a well-known lectin that binds glucose [8], whereas jacalin is a prototype representative of the jacalin-related lectin family that binds galactose [9].

Next, we describe how the binding of glucose to its receptor can be verified using an In-cell ELISA with CHO-K cells stably expressing the recently identified glucose receptor ADGRL1 [6]. Further, we demonstrate how to determine the binding kinetics and interaction between glucose and ADGRL1 using the surface plasmon resonance (SPR) technique. We used a Biacore Sensor Chip that immobilizes different ligands including carbohydrates to measure their kinetics of binding and affinity with their receptors or binders [10, 11].

## Protocol

### Immobilization of glucose– (or other carbohydrate–) biotin–PAA conjugates to enrich glucose or carbohydrate-binding receptor/s

#### Reagents

**Figure 1.**
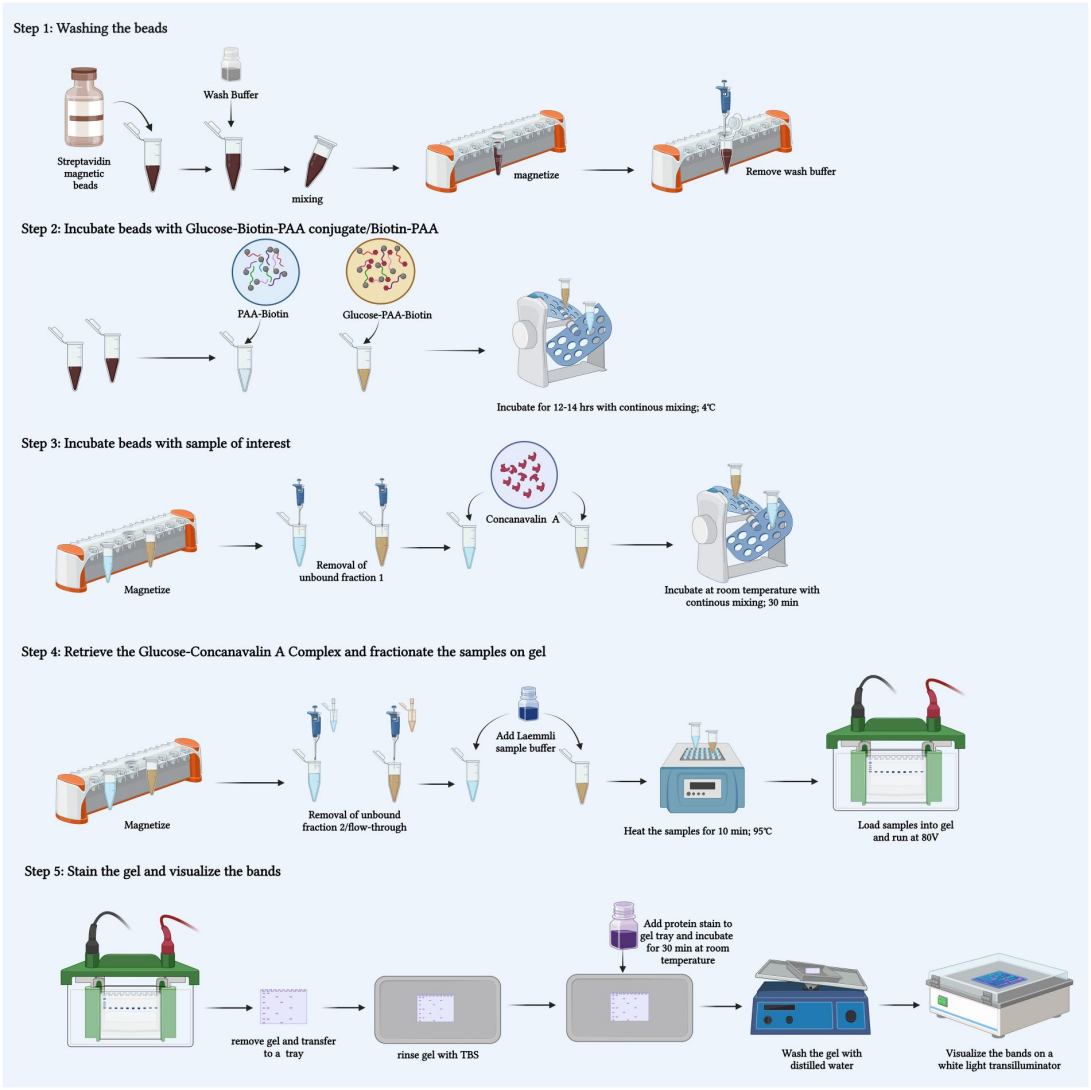
Step-wise protocol for immobilization of glucose–biotin–PAA conjugate onto streptavidin magnetic beads, enrichment and retrieval of glucose-concanavalin A complex followed by separation and visualization of retrieved proteins onto polyacrylamide gel (Figure was made using Biorender.com)

1. Pierce™ Streptavidin Magnetic Beads (catalog no: 88816/88817)
2. 10× Tris-buffered saline (TBS) (Bio-Rad, catalog no #1706435)
3. Binding/Wash Buffer (TBST): TBS containing 0.1% (v/v) Tween™ 20 Detergent
4. Elution buffer: 2× Laemmli buffer (Bio-Rad, catalog no: #1610737)
5. Biotinylated glucose or carbohydrate conjugates (custom synthesis by GlycoNZ, New Zealand) [12]
6. Proteins or samples of interest
7. Mini-PROTEAN^®^ TGX™ Precast Gels 4-20% (Bio-Rad, catalog no: #4561094)
8. Imperial™ Protein Stain (Thermo Scientific™, catalog no: 24615)
9. Precision Plus Protein WesternC Standards (Bio-Rad, catalog no: #161-0376) (Fig. 1)

#### Equipment setup

1. Magnetic rack (New England Biolabs, Inc., catalog no: S1509S)
2. Microtube rotator
3. Dry block heater
4. Mini-PROTEAN Tetra Cell (Bio-Rad, catalog no-552BR)
5. Rocking shaker
6. 1.5- or 2-ml microcentrifuge tubes
7. Micropipettes
8. Microtips

#### Procedure

1. Washing the magnetic beads: Take 75 µl of Pierce Streptavidin Magnetic Beads in a 2 ml microcentrifuge tube. Add 1 ml of TBST wash buffer to the beads and invert the tube three times or vortex gently to mix the beads. Place the tube into a magnetic rack and allow the beads to separate from the supernatant. Remove the supernatant (wash buffer) and discard it. Take the tube out of the magnetic rack and wash the beads again with 1 ml TBST. Separate the beads in the magnetic rack and then remove the supernatant.
2. Immobilization of glucose– (or other carbohydrate–) biotin–PAA conjugate: Add 1 ml of PAA-biotin (control) and 1 ml of glucose– (or other carbohydrate–) biotin–PAA (1 mg/ml) to the respective streptavidin magnetic bead tubes and incubate overnight (continuously mixing using a microtube rotator, at 4°C). Next, take the tubes out of the rotator and place them into a magnetic rack to separate the beads and remove the supernatant (unbound fraction 1). Then wash the beads twice with TBST.
3. Enrichment of glucose- or other carbohydrate-binding protein/receptor: Incubate the beads with 20 µg/ml of the sample of interest (concanavalin A or jacalin are used in this study) in 500 µl TBS buffer at room temperature for 30 min with continuous mixing using a rotator. Then separate the beads using a magnetic rack and remove the supernatant (unbound fraction 2/flow-through).
4. Retrieval of enriched receptor/s bound to glucose– (or other carbohydrate–) biotin–PAA conjugate: Elute the bound fraction from the beads by adding 25 µl of 2× Laemmli buffer to the beads. Use 5 µl of concanavalin A (1 mg/ml) mixed with an equal volume of 2× Laemmli buffer as a positive control. Add equal volume of 2× Laemmli buffer to flow-through/unbound fraction 2 described in step (3). Heat all the samples at 95°C in a dry-block heater for 10 min. Then allow the samples to cool at room temperature for 5 min before loading them into the wells of the precast gel.
5. Separation of the enriched proteins using SDS-PAGE (sodium dodecyl sulfate-polyacrylamide gel electrophoresis): Take the Mini-PROTEAN^®^ TGX™ Precast Gel (4%–20%) and place it in a gel cassette in Mini-PROTEAN Tetra Cell. Load the samples into respective wells and use Precision Plus Protein™ WesternC™ Blotting Standard as a molecular-weight ladder. Connect the Tetra Cell with the power supply and run the gel at 80 V for 1 h. Once the gel run is complete, remove the gel from its cassette and place it into a tray containing 1× TBS. Rinse the gel in TBS for 2 min before transferring the gel into a tray containing Imperial Protein Stain. Stain the gel for 30 min at room temperature according to manufacturers’ instructions. Once the gel is stained, transfer it to a tray containing distilled water. Remove the excess background stain on the gel by gentle shaking the tray on a rocker for 1 h. The water may be replaced with fresh distilled water every 20 min during the shaking to completely remove the background stain. Visualize the stained protein bands by placing the gel on a white light trans-illuminator.
6. Identification of enriched glucose- or other carbohydrate-binding receptor/s: To identify the retrieved and enriched glucose- or other carbohydrate-binding receptor/s, the desired protein bands can be excised from the gel and identified through a mass spectrometry-based proteomics technique as described in Shevchenko *et al*. [13].

#### Precautions

- Avoid introducing bubbles at any pipetting step as this may lead to loss of beads and sample.
- Allow the suspension containing beads to be on the magnetic rack for at least a minute before removing the supernatant. Cross check visually also whether beads have settled down and moved out of suspension.
- Don’t disturb the pellet, and keep the tubes in the magnetic rack while removing the supernatant.

### Verification of binding of glucose to its putative receptor using In-cell ELISA

#### Reagents

**Figure 2.**
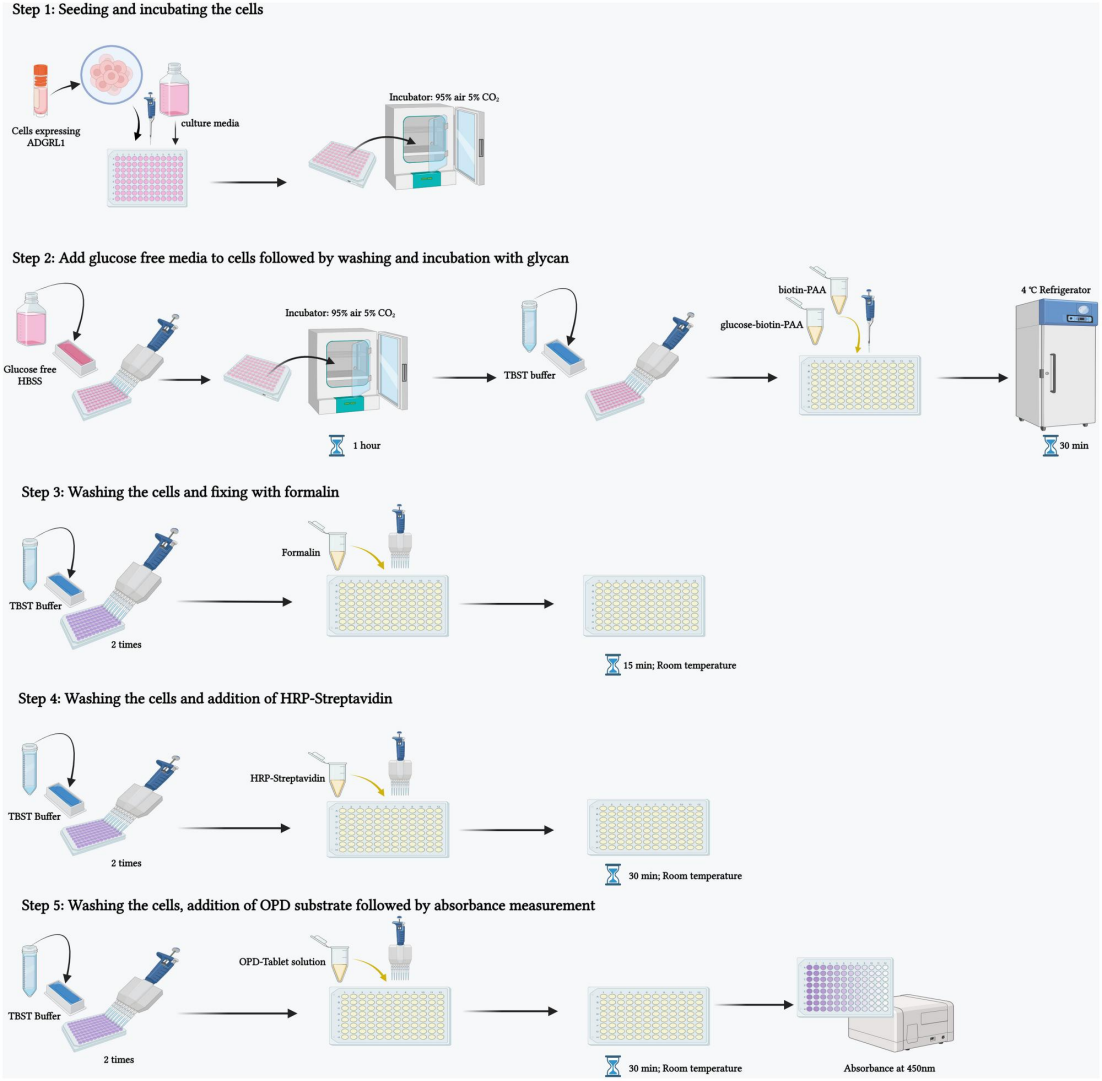
Step-wise protocol for In-cell ELISA to confirm the binding of glucose to its receptor ADGRL1 (Figure was made using Biorender.com)

1. Cell line/s (expressing putative glucose receptor)
2. Glucose-free Hank’s Balanced Salt Solution (HBSS [14], glucose was not added to the buffer)
3. 10× TBS (Bio-Rad, catalog no: #1706435)
4. 1× TBS containing 0.1% (v/v) Tween™-20 Detergent (TBST)
5. Methanol free formaldehyde (Thermo Scientific, catalog no: 28906)
6. Horseradish peroxidase–Streptavidin (HRP-Streptavidin) conjugate (Thermo Scientific, catalog no: SA10001)
7. O-Phenylenediamine hydrochloride (OPD) Tablets (Thermo Scientific, catalog no: 34006)
8. Peroxidase buffer (Sigma, catalog no: H1009) (Fig. 2)

#### Equipment setup

1. Laminar flow hood
2. CO_2_ incubator
3. Refrigerator or cold storage room
4. Spectrophotometer
5. 96-well cell culture clear bottom microplates
6. Disposable reagent reservoirs
7. Micropipettes
8. Microtips

#### Procedure

1. Culture CHO-K cells expressing a putative glucose receptor in a 96-well plate (∼10 000 cells/well) in a suitable medium. On the day of experiment, replace the cell culture medium with glucose-free HBSS and incubate for 1 h in a cell culture incubator (95% air 5% CO_2;_ 37°C).
2. Wash the cells with TBST buffer and then incubate with glucose–biotin–PAA or control conjugates (1 mg/ml) for 30 min at 4°C in a refrigerator or cold room.
3. After the incubation, wash the cells twice with TBST buffer.
4. Fix the cells with 4% formaldehyde for 15 min at room temperature.
5. Wash the cells twice with TBST buffer. Prepare HRP-streptavidin (1:10 000) in glucose free HBSS and add the solution to the wells. Incubate the cells for 30 min at room temperature.
6. After the incubation, wash the cells twice with TBST and then add OPD tablet solution prepared in 1× peroxidase buffer to the wells. Incubate the cells for 20–30 min at room temperature.
7. Measure absorbance at 450 nm within 15 min of adding the OPD solution using a spectrophotometer.

#### Precautions

- Don’t let the plate dry at any step of the assay.
- Use a new disposable reagent reservoir at every step while using multichannel pipette.

### Assessment of the binding kinetics and interaction between glucose and its receptor using SPR analysis

#### Reagents

**Figure 3.**
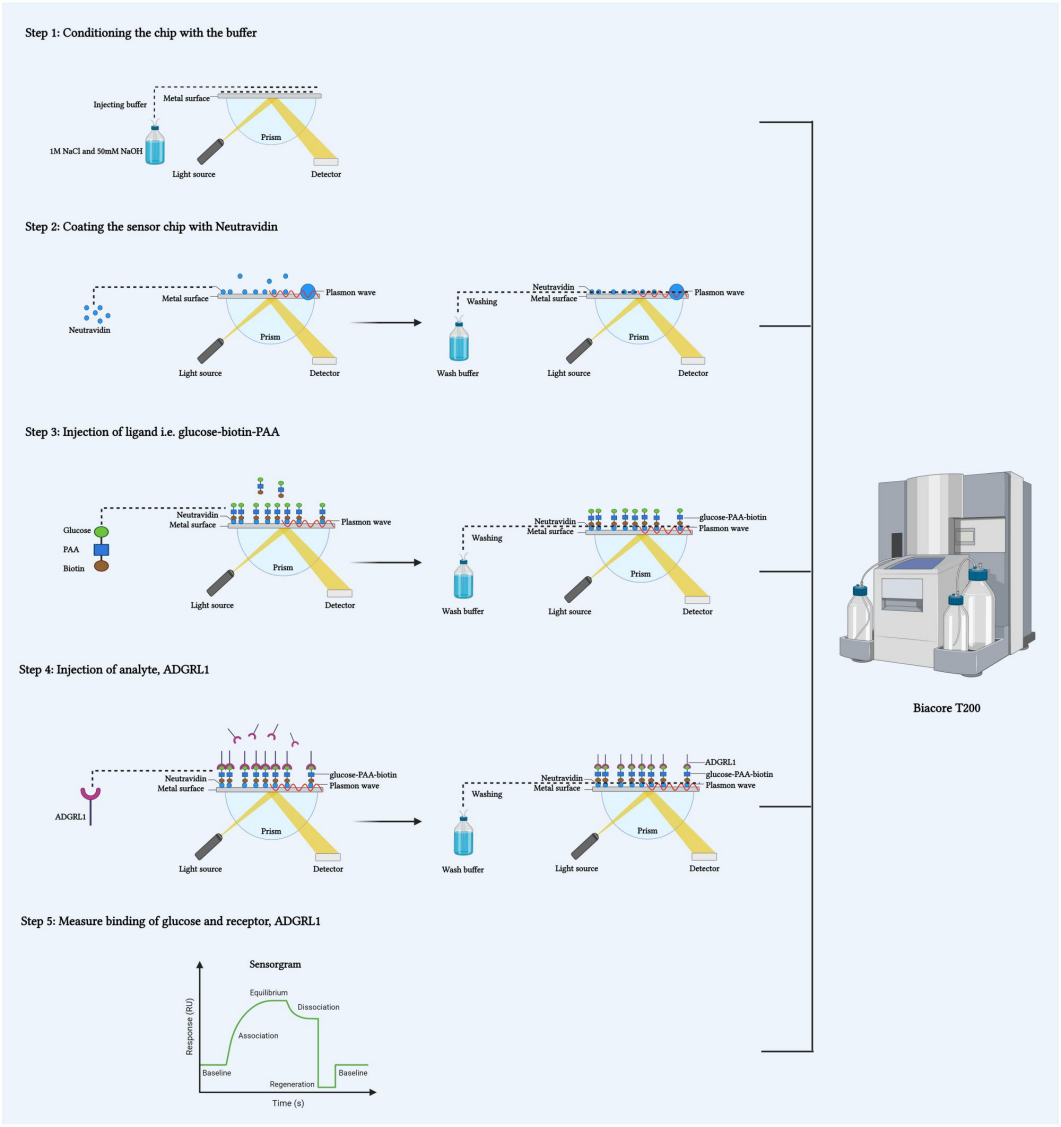
Step-wise protocol for validating the binding of glucose to its receptor ADGRL1 through SPR (Figure was made using Biorender.com)

1. Biacore Series S sensor chip NA (Cytiva, catalog no: 29699622)
2. SPR Running buffer (10× HBS-P+) (Cytiva, catalog no: BR100671)
3. Tween 20 (0.05% (v/v))
4. Dimethyl sulfoxide DMSO (10% (v/v) concentration)
5. Analyte sample (prepared in running buffer)
6. 1 M sodium chloride (NaCl)
7. 50 mM sodium hydroxide (NaOH) (Fig. 3)

#### Equipment setup

1. Biacore T200 (Cytiva)
2. Reagent well plates
3. Microplate foil to cover
4. Reagent tubes and caps
5. Micropipettes
6. Microtips

#### Procedure (refer to Cytiva.com/Biacore for further optimization of the protocol and method)

1. Equilibrate the sealed sensor chip at room temperature for 15–30 min. Meanwhile prepare the Biacore instrument with the running buffer per the instrument’s manual.
2. Open the sensor chip and dock it in the instrument (per the instrument’s manual)
3. Condition the sensor surface with three consecutive 1-min injections of 1M NaCl and 50 mM NaOH before ligand immobilization.
4. Then, allow the chip to equilibrate in running buffer for 10 min.
5. Immobilize different concentrations of ligand (e.g. Glucose–PAA–Biotin) at a flow rate of 10 µl/min onto the Sensor Chip to achieve the desired resonance units (RU). Reference flow cell has no immobilized ligand.
6. Run the kinetic/affinity experiment overnight at a flow rate of 30 µl/min using 1:2 dilution of analyte (e.g. Adgrl1; 2000–125 nM concentration).
7. Measure the binding with at least three different concentrations of ligands or analytes.

#### Precautions

- Equilibrate the sensor surface completely to avoid baseline drift.
- Check for mismatch of running and analyte buffers to avoid shift in the curve at the beginning and end of the experiment.
- Check for non-specific binding of the analyte to the reference surface using blank reference surface and blank sample injection.

## Results

**Figure 4.**
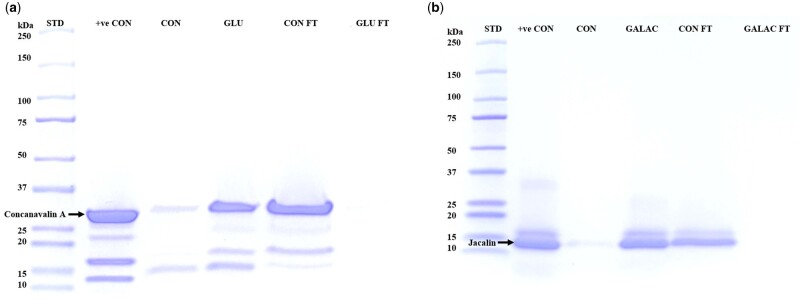
Demonstration of binding of glucose with concanavalin A and galactose with jacalin using affinity chromatography (streptavidin magnetic beads and glucose conjugates) followed by SDS-PAGE. (a) 4–20% Polyacrylamide gel stained with Imperial Protein Stain. STD: protein standard; +ve CON: concanavalin A; CON: Biotin–PAA immobilized onto streptavidin magnetic beads; GLU: glucose-biotin–PAA immobilized onto streptavidin magnetic beads; CON FT: Control flow-through (supernatant); GLU FT: glucose flow-through (supernatant) (b) +ve CON: jacalin; CON: biotin–PAA immobilized onto streptavidin magnetic beads; GALAC: galactose–biotin–PAA immobilized onto streptavidin magnetic beads; CON FT: control flow-through (supernatant); GALAC FT: galactose flow-through (supernatant). The non-specific bands represent streptavidin leaching from the magnetic beads

Retrieval of enriched concanavalin A or jacalin using immobilized glucose or galactose–biotin–PAA conjugates onto streptavidin magnetic beads:

Streptavidin magnetic beads coated with PAA-biotin (control; no carbohydrate) and subsequently incubated with concanavalin A (a known glucose-binding protein), showed no protein bands indicative of no binding of concanavalin A due to the absence of glucose moiety (CON in Fig. 4a). This observation was further confirmed by the presence of concanavalin A band corresponding to 30 kDa in the flow through (fraction unbound to the conjugate-coated magnetic beads) solution collected from the control group (CON FT in Fig. 4a). In contrast, streptavidin magnetic beads coated with glucose–biotin–PAA conjugate bound concanavalin A as shown by the band around 30 kDa (GLU in Fig. 4a) which matched with the positive control (+ve CON in Fig. 4a). As expected, no concanavalin A band was observed in the flow through because the protein was bound to the beads (GLU FT in Fig. 4a).

Similarly, when streptavidin magnetic beads coated with PAA-biotin (control, no carbohydrate) were incubated with jacalin (a known galactose-binding protein), there was no binding observed (CON in Fig. 4b) due to absence of galactose moiety and therefore jacalin was washed out with the flow through indicated by band 14 kDa (CON FT in Fig. 4b). Streptavidin magnetic beads coated with galactose–biotin–PAA conjugate bound jacalin as shown by the presence of its band at 14 kDa (GALAC in Fig. 4b), which matched the positive control. This observation was further validated by absence of jacalin in the flow through (GALAC FT in Fig. 4b).

**Figure 5.**
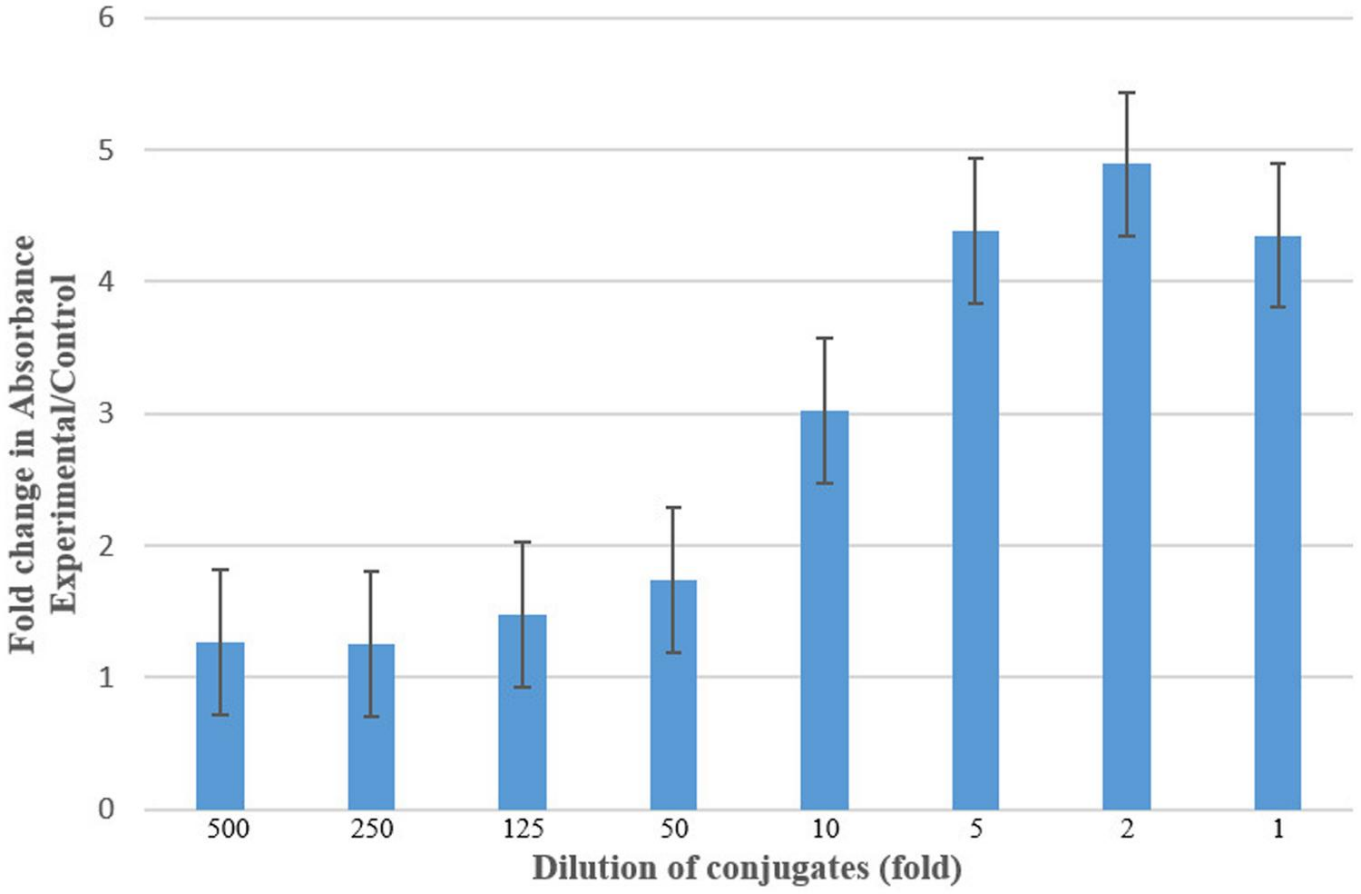
In-cell ELISA. Binding of adhesion G-protein coupled receptor L1 (ADGRL1) (which was stably expressed in CHO-K cells) to glucose–biotin–PAA conjugate or its control. Experimental: glucose–biotin–PAA; control: biotin–PAA. The data are presented as mean  ±  SEM from four replicates

Verification of the binding of glucose with ADGRL1 using In-cell ELISA:

CHO-K cells showing stable expression of hADGRL1 were seeded in 96-well cell-culture plates and cultured. Cells were then incubated with different concentrations of glucose–biotin–PAA conjugate (stock: 4 mg/ml) and control, namely biotin–PAA (stock: 2 mg/ml). This step allowed binding of glucose–biotin–PAA conjugate to the receptor ADGRL1 expressed by the cells. After fixing and washing the cells, cells were then incubated with HRP-Streptavidin to detect the biotin present in glucose–biotin–PAA conjugate, which is bound to ADGRL1. On addition of the chromogenic substrate OPD for HRP, there was formation of colored product which was measured by absorbance at 450 nm. Absorbance values were progressively higher (1.27, 1.25, 1.48, 1.73, 3.02, 4.38, 4.89, 4.35 fold GLU versus CON) with an increase in the concentration (1:500, 1:250, 1:125, 1:50, 1:10, 1:5, 1:2, 1-fold dilution) of glucose–biotin–PAA conjugate (stock: 4 mg/ml) (Fig. 5).

**Figure 6.**
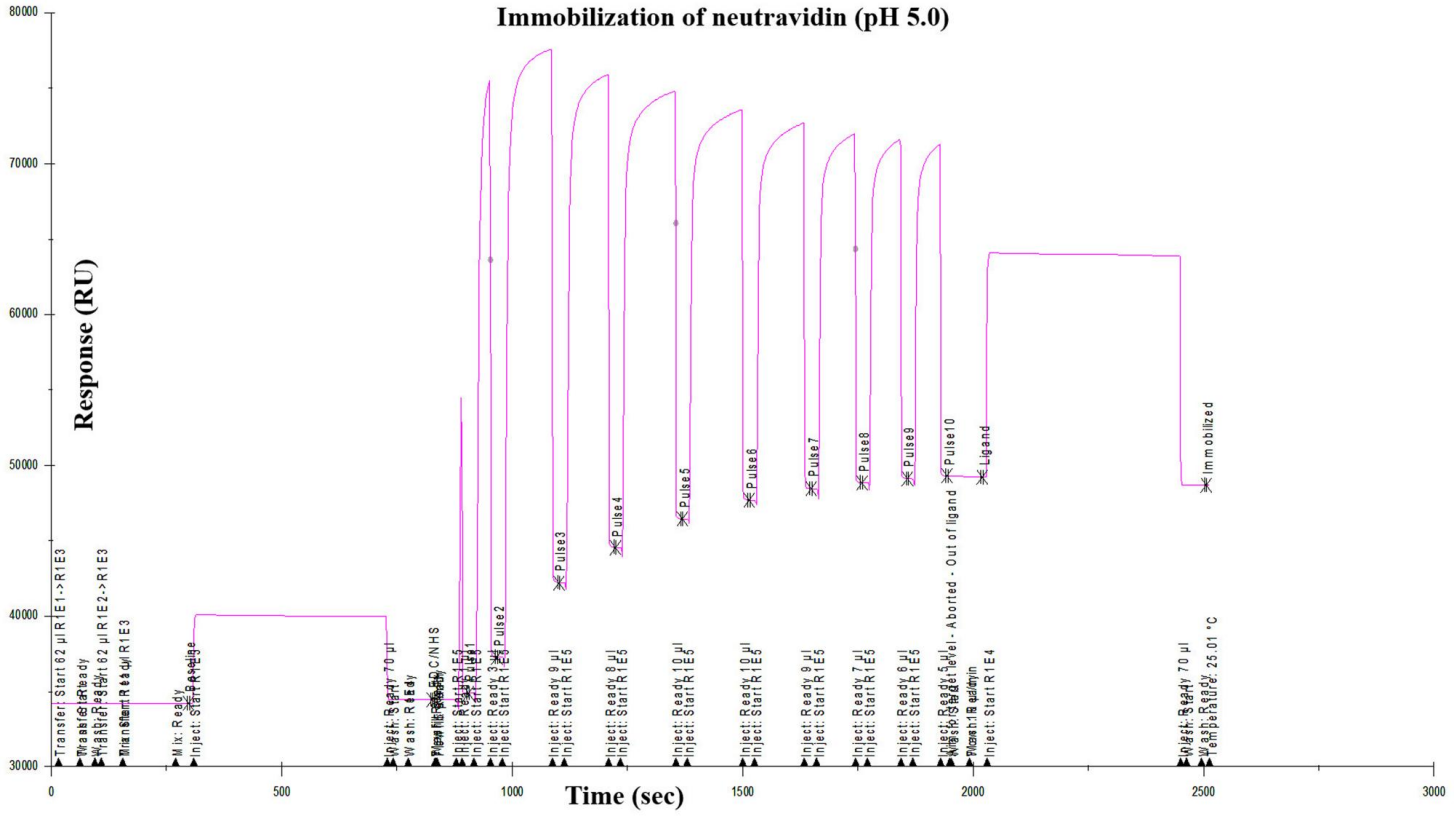
Immobilization of neutravidin onto the surface of the Biacore sensor chip. RU, resonance units

**Figure 7.**
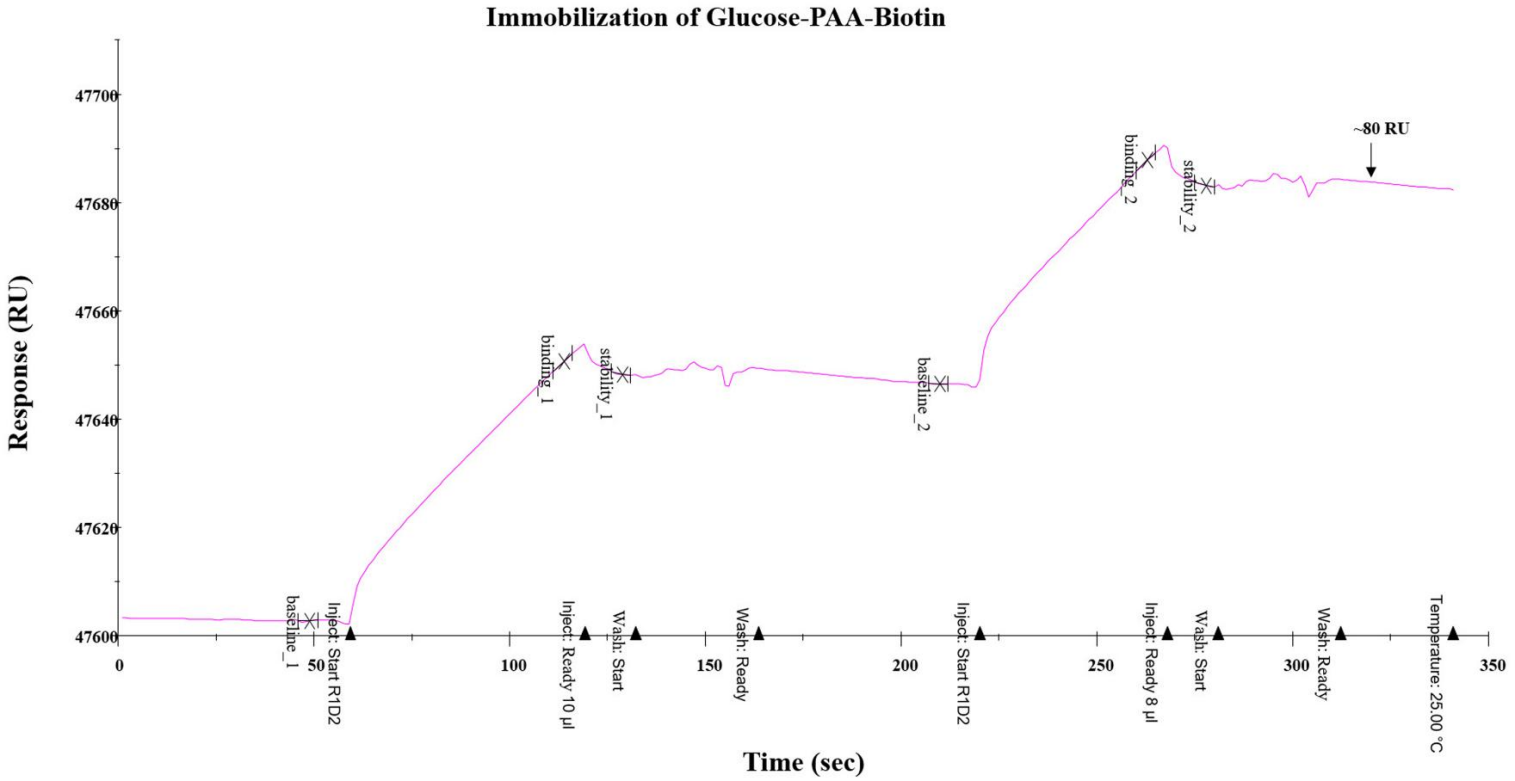
Immobilization of glucose-biotin-PAA onto the neutravidin-coated Biacore sensor chip using biotin-neutravidin interaction. RU, resonance units

**Figure 8.**
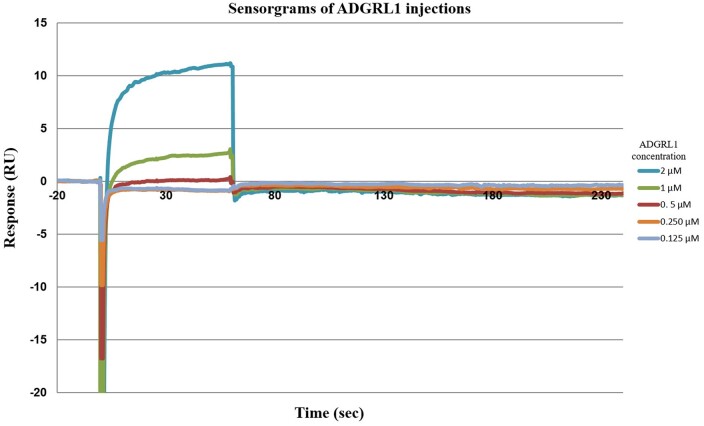
Sensorgrams of ADGRL1 injections. RU, resonance units

Detection and binding kinetics of glucose with receptor ADGRL1:

Using the Biacore Series S sensor chip coated with neutravidin (15 000 RU, Fig. 6), glucose-PAA-biotin was immobilized using the neutravidin-biotin interaction. The RU was further increased by 80 units on injecting additional glucose-PAA-biotin (Fig. 7). After rinsing the sensor surface, recombinant ADGRL1 was then injected (0.125, 0.250, 0.5, 1, and 2 µM conc in TBS) at 60 and 90 s intervals at flow rate of 30 ul/min, which led to an increase of RU by 12 units at 2 µM conc (Fig. 8). After each binding or coating step, the achieved RU was set to baseline (0) before additional ligand or analytes were flowed over the chip surface.

## Discussion

In this protocol, we have shown how a glucose- or carbohydrate-binding receptor can be isolated from samples of interest such as tissue homogenates or single-cell suspensions. The isolated receptor can then be identified through mass spectrometry-based proteomics and further verified using highly sensitive SPR. We immobilized glucose–biotin–PAA or galactose–biotin–PAA conjugates onto streptavidin-coated magnetic beads, followed by incubation of these beads with concanavalin A or jacalin solution to demonstrate how the glucose conjugates can enrich their known binding proteins. This protocol can be extended to any biotin-labeled polyacrylamide-conjugated glycan to isolate the corresponding glycan-binding receptors from tissue lysates and single-cell suspensions. In addition, the protocol can be optimized to enrich non-protein glucose or carbohydrate binders such as glycoRNAs [15] followed by an appropriate technique of interest to identify the binders. Use of magnetic beads provides several advantages over conventional agarose or Sepharose beads to isolate carbohydrate-binding receptors using lectin affinity chromatography [16]. For example, agarose is a carbohydrate-derived porous material and may therefore increase non-specific binding. In addition, with magnetic beads, diverse cell samples with large volumes can be processed with relatively high throughput using multiple magnetic racks. The smaller size and non-porous material of these beads present a greater surface area for sample binding and less void volume. These beads also allow fast processing times and low sample loss, with an easy cleanup process after completing the protocol. Finally, these magnetic beads provide an added advantage of enhancing specificity and reducing noise by being able to be pre-conjugated with desired molecules (including carbohydrates) and then isolating proteins or receptors from samples of interest.

Once the carbohydrate-binding receptor/s are identified, stable cell line/s can be produced to express those receptors and establish their interactions*.* This system can be further utilized to study the downstream signaling mechanism of the glycan–receptor interactions and, from this high-throughput screening for drug discovery. For in-depth analysis of carbohydrate–receptor interaction, we recommend the use of the SPR technique. We recently used both In-cell ELISA and SPR technique to validate glucose–ADGRL1 interactions [6].The SPR method not only allows to confirm glycan–receptor binding but also enables the user to determine the binding kinetics simultaneously. This is crucial to determine the affinity of the receptor toward glycan at low concentrations.

Up to the present, biochemists have attempted to synthetically design glucose receptors using computational approaches to detect glucose levels and to develop glucose-based assays for diagnostic- and bioprocess-monitoring purposes [17–19]. However, methods or protocols to identify glucose- or carbohydrate-binding receptors in mammalian systems remain scarce. The conventional lectin affinity chromatography is mainly used to isolate glycoproteins and glycolipids, but the method is not sensitive enough to directly isolate carbohydrate-binding receptors.

The protocol described here has potential for identifying novel glucose- or carbohydrate-binding receptors. Identification of these receptors will help in determining the signaling pathways through which these nutrients are sensed in the body both in health and in disease. The information could also be utilized to develop efficient glucose-sensing systems and devices as well as alternatives to insulin therapy with better precision and efficient monitoring. The glucose receptors can be targeted for treatment of metabolic diseases such as diabetes and its complications or glycosylation related disorders. Apart from this, identification of glucose or carbohydrate-binding receptors can have wide applications from identification of pathogen recognition to cell adhesion, extravasation and metastatic processes [20]. Identification of such receptors will also provide deeper insights about glycoproteins in circulation and can be exploited for development of effective therapeutics for glycoprotein clearance such as in Gaucher’s disease [21–23]. Recently, lectin receptors conserved for SARS-CoV-2 glycosylation sites were identified and that revealed receptors involved in spike binding of a SARS-CoV-2 variant [24]. This further emphasizes the importance and wide applicability of glucose- or carbohydrate-receptors in biomedical research and medicine.

## Limitations

1. Multiple glucose or other saccharide receptors with different affinities are expected to be discovered using this protocol. Therefore, subsequent verification steps using In-cell ELISA and SPR will be necessary to validate the receptors with highest affinities. For example, galactose doesn’t bind to concanavalin A [25] (which is known to predominantly bind glucose and mannose), but glucose and mannose at high concentrations may bind to jacalin [9] (which is known to predominantly bind galactose).
2. Non-specific binding of magnetic beads with natural iron-binding proteins in the samples of interest should be considered in the study design.
3. Biotin–polyacrylamide itself can potentially also lead to non-specific binding. This concern can be addressed using appropriate controls.
4. Expressing the identified receptor/s (especially high molecular weight receptors) in suitable cell line/s can be a challenge and may require an advanced expertise in molecular and cell biology.
5. Solubilizing and injecting high concentrations of receptor when using SPR equipment can be a limitation.
6. Magnetic beads are relatively costly compared to agarose beads.

## Author contributions

Nadia Rashid (Conceptualization [equal], Data curation [equal], Formal analysis [equal], Investigation [equal], Methodology [equal], Validation [equal], Writing—original draft [equal], Writing—review & editing [equal]) and Kavaljit H. Chhabra (Conceptualization [equal], Data curation [equal], Formal analysis [equal], Funding acquisition [equal], Investigation [equal], Methodology [equal], Project administration [equal], Resources [equal], Supervision [equal], Writing—original draft [equal], Writing—review & editing [equal])

*Conflict of interest statement.* Authors have no conflict of interest.

## Data and materials availability

All data are available in the main text. The reagents used in this study are available via material transfer agreement addressed to the corresponding author.
